# Supplementary material for: Nuclear numbers in syncytial muscle fibers promote size but limit the development of larger myonuclear domains
Source: Nat Commun. 2020 Dec 8;11:6287. doi: 10.1038/s41467-020-20058-7 (PMC7722938; doi:10.1038/s41467-020-20058-7)
Supplement: Supplementary file 1 — Supplementary Information [file 41467_2020_20058_MOESM1_ESM.pdf]

Supplementary Information

Nuclear numbers in syncytial muscle fibers promote size but limit flexibility

Cramer AW. et al.

This PDF file includes:

Supplementary Figure 1
Supplementary Figure 2
Supplementary Figure 3
Supplementary Figure 4
Supplementary Figure 5
Supplementary Figure 6
Supplementary Figure 7

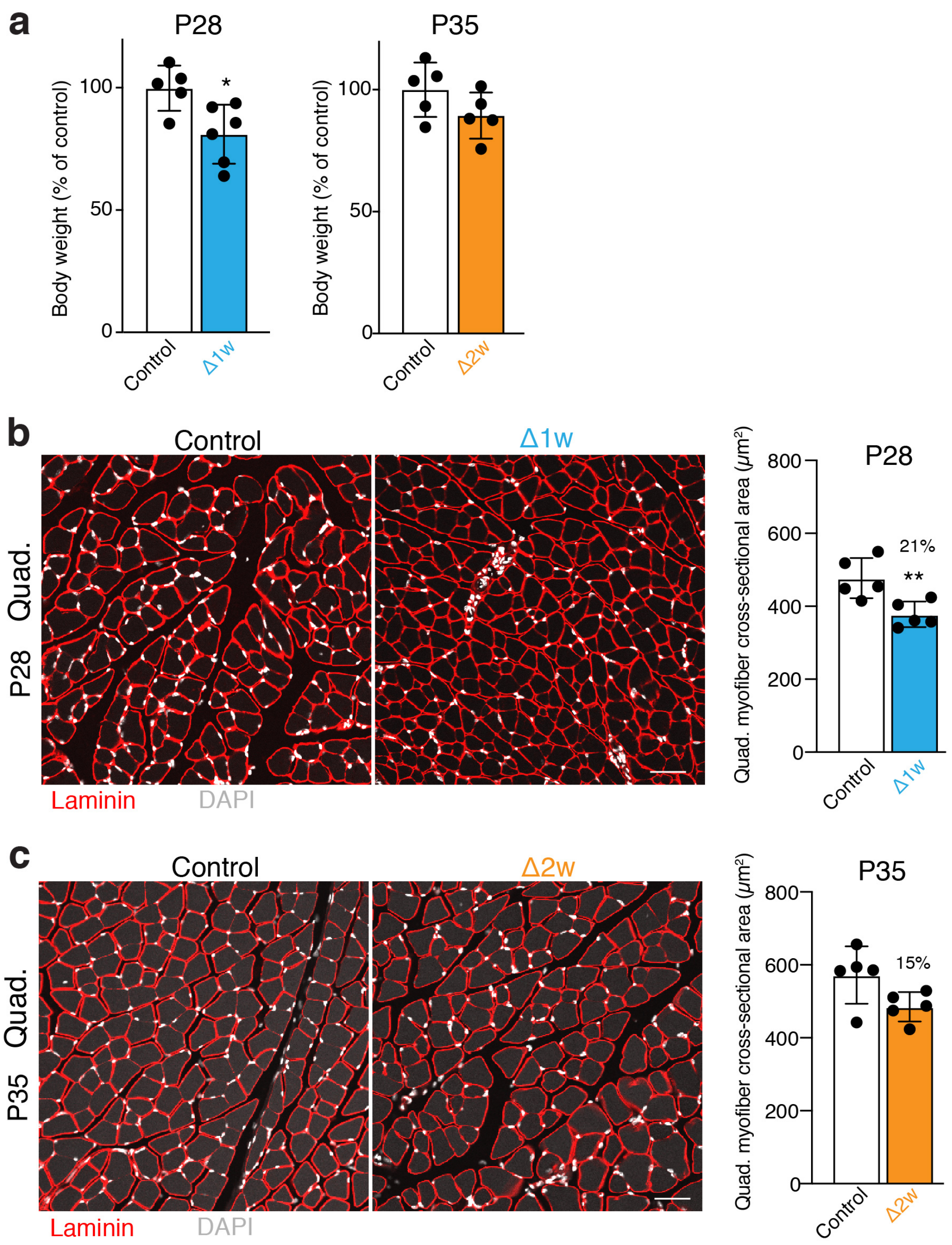

Supplementary Fig. 1 Effects of reduced myonuclear number on cross-sectional area of quadriceps. **a** Body weights of Δ1w (left) and Δ2w (right) mice expressed as a percent of their respective control mice 4 weeks after tamoxifen administration (n = 5-6 biologically independent animals). **b** Sections of quadriceps (Quad.) muscle of Δ1w and control mice at P28 (left). Analysis of cross-sectional area (right) revealed a 21% reduction in Δ1w myofiber cross-sectional area compared to controls. **c** Sections of quadriceps muscle of Δ2w and control mice at P35 (left). Cross-sectional area analysis (right) showed Δ2w myofibers are 15% smaller than controls in the quad. Sections in **b** and **c** were immunostained with laminin antibodies (red) and stained with DAPI (white). At least 3 20x images were analyzed per mouse in **b** and **c** (n = 5 biologically independent animals). Statistical analyses and data presentation: **a-c**, two-sided unpaired *t*-test; *p<0.05,

**p<0.01. Data are represented as mean ± SD. Scale bar: 50 µm. Source data are provided as a Source Data file.

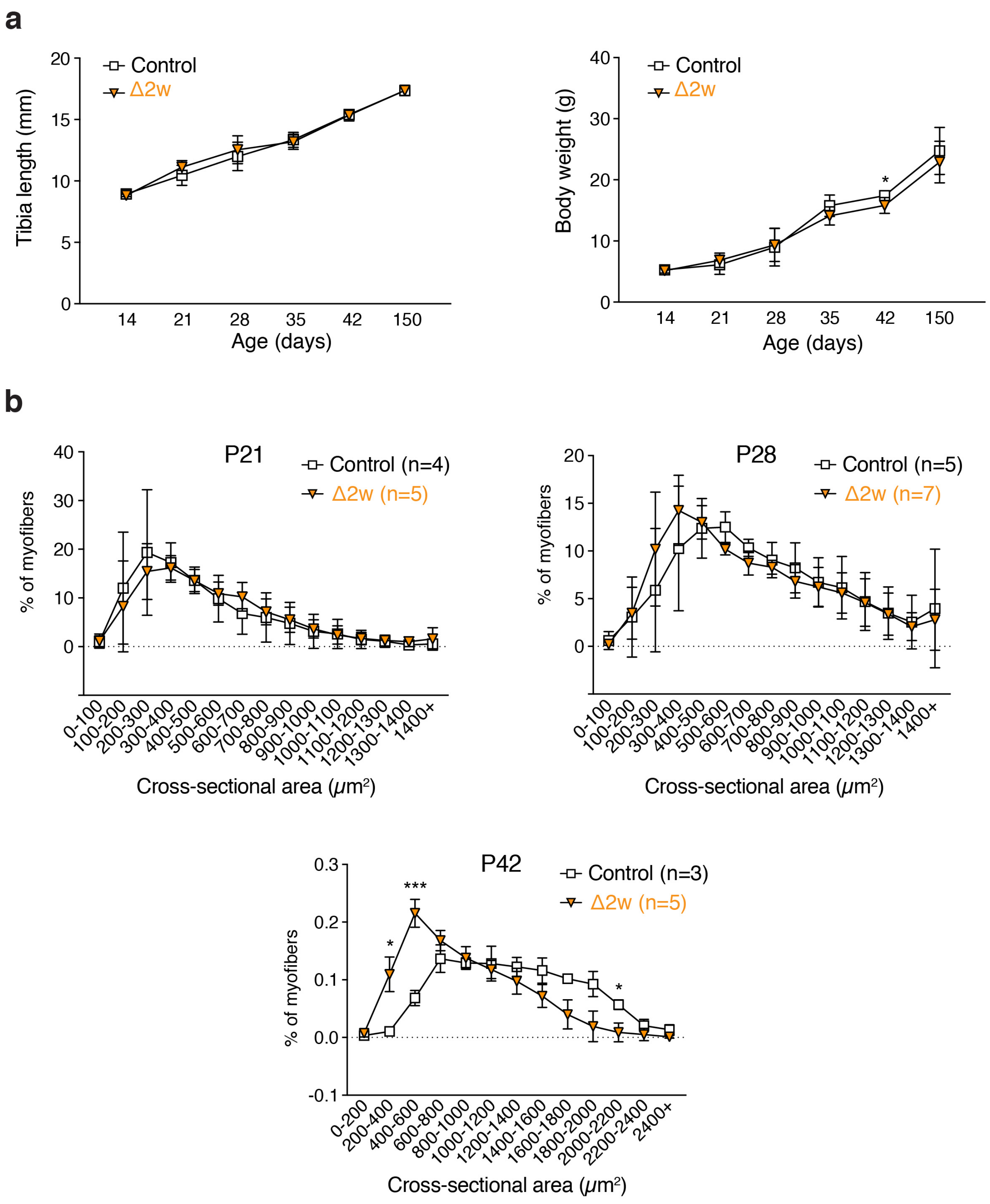

Supplementary Fig. 2 Kinetics of muscle growth in Δ2w mice. **a** Tibia length (left) and body weight (right) measurements at various timepoints revealed Δ2w mice grow at the same rate as controls from P14 to P150 (P14 control n=3, Δ2w n=4; P21 control n=6, Δ2w

n=10; P28 control n=6, Δ2w n=8; P35 control n=5, Δ2w n=5; P42 control n=7, Δ2w n=9; P150 control n=5, Δ2w n=15). **b** Tibialis anterior myofiber cross-sectional area size distribution at P21, P28, and P42. At least 3 20x images were analyzed per mouse. Statistical analyses and data presentation: **a** and **b** multiple two-sided unpaired *t*-tests (**a**: one per timepoint, **b**: one per CSA bin); *p<0.05, ***p<0.001. Data are represented as mean ± SD. Source data are provided as a Source Data file.

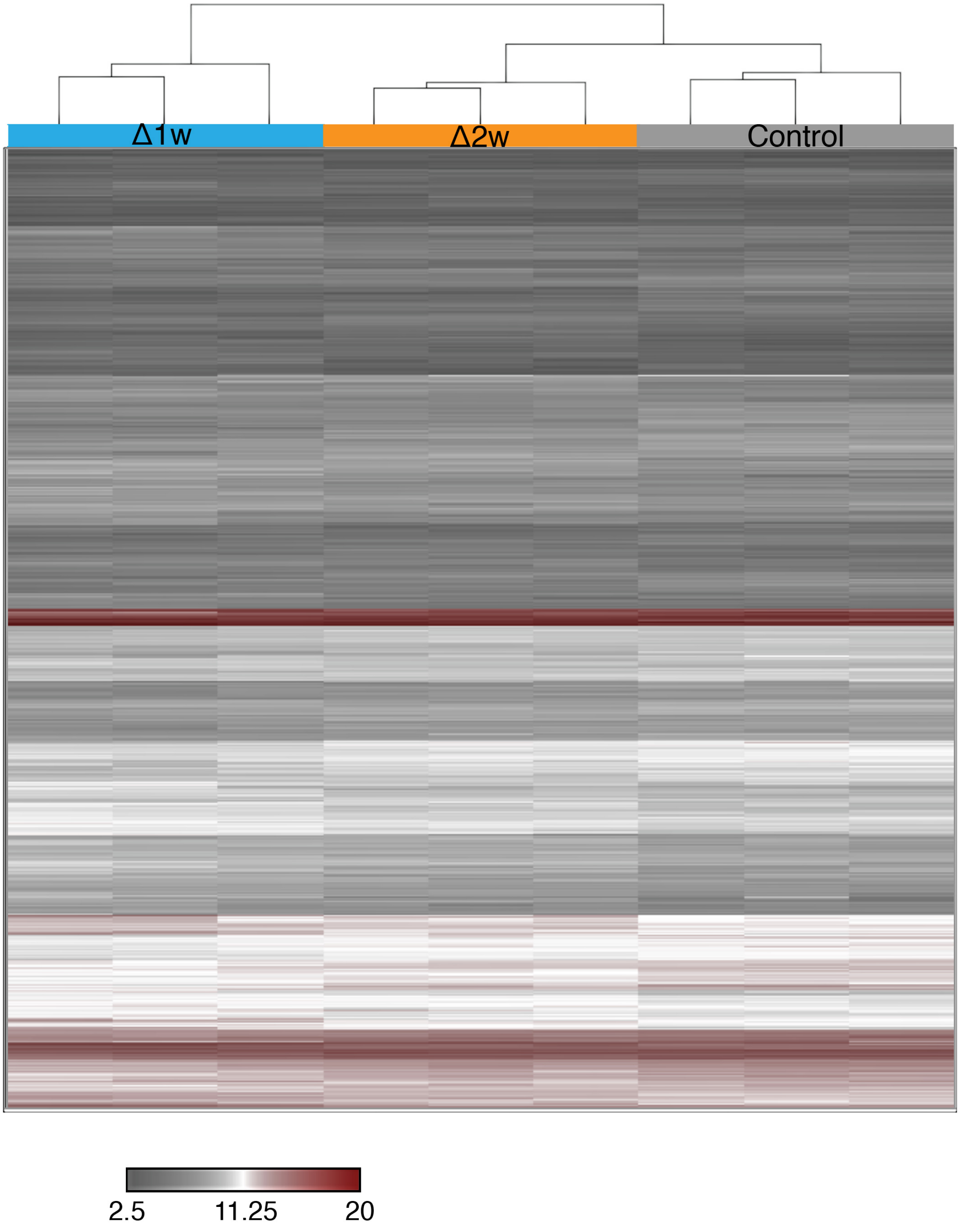

Supplementary Fig. 3 Hierarchichal cluster analysis of control, Δ1w, and Δ2w muscle at P28.

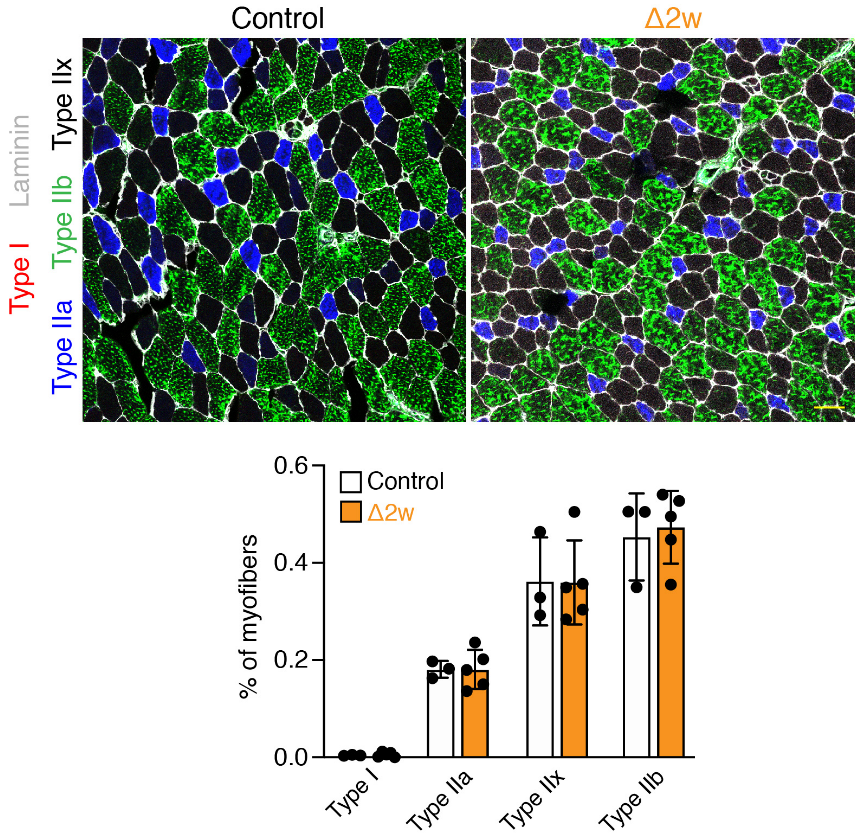

Supplementary Fig. 4 Tibialis anterior fiber type distribution in Δ2w muscle. Sections of tibialis anterior (TA) muscle in control and Δ2w mice at 5-7 months of age immunostained with antibodies for MYH7 (Type I; red), MYH2 (Type IIa; blue), MYH4 (Type IIb; green), and laminin (white) antibodies. Unstained fibers were counted as Type IIx myofibers. 3 20x images were analyzed per mouse (n = 3-5 biologically independent animals). Data presentation: Data are represented as mean ± SD. Scale bar: 50 µm. Source data are provided as a Source Data file.

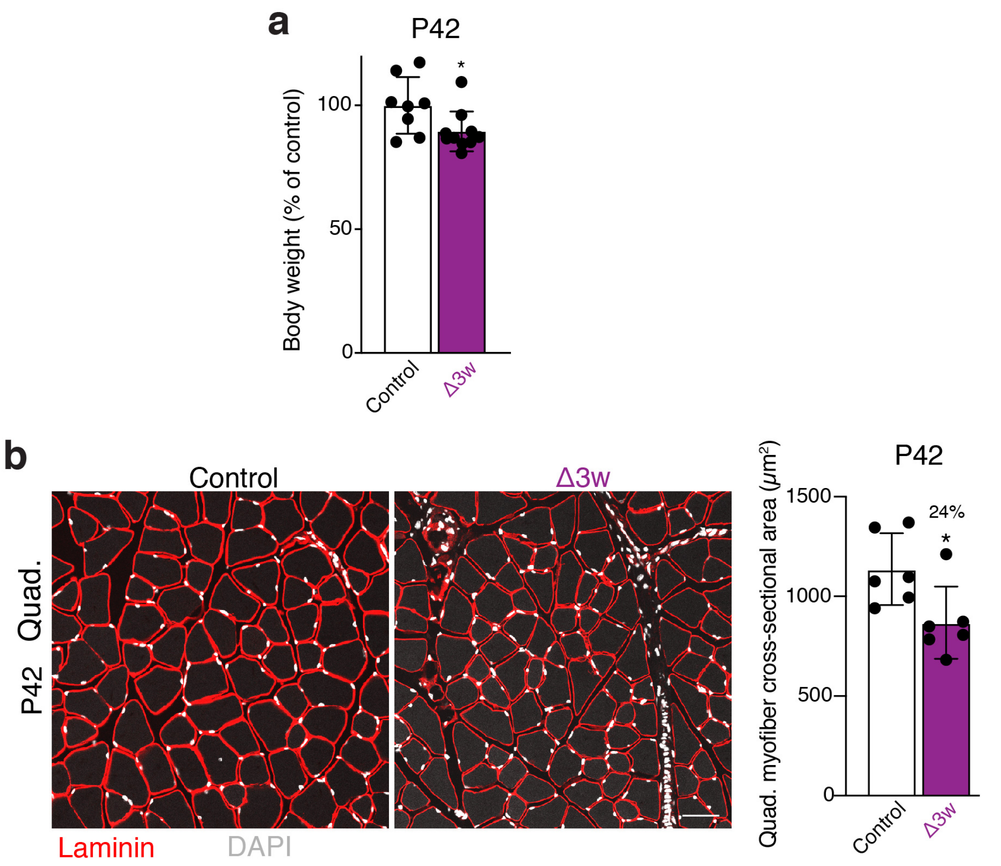

Supplementary Fig. 5 Effects of reduced myonuclear number in Δ3w muscle. **a** Body weight of Δ3w mice expressed as a percent of control at P42 (n = 8-9 biologically independent animals). **b** Cross-sections of quadriceps (Quad.) muscle of Δ3w and control mice at P42 (left). Cross-sectional area analysis (right) reveals a 24% reduction in Δ3w quadriceps compared to controls. Sections were immunostained with laminin antibodies (red) and stained with DAPI (white). At least 3 20x images were analyzed per mouse (n = 6 biologically independent animals). Statistical analyses and data presentation: **a** and **b** two-sided unpaired *t*-test; *p<0.05. Data are represented as mean ± SD. Scale bar: 50 μm. Source data are provided as a Source Data file.

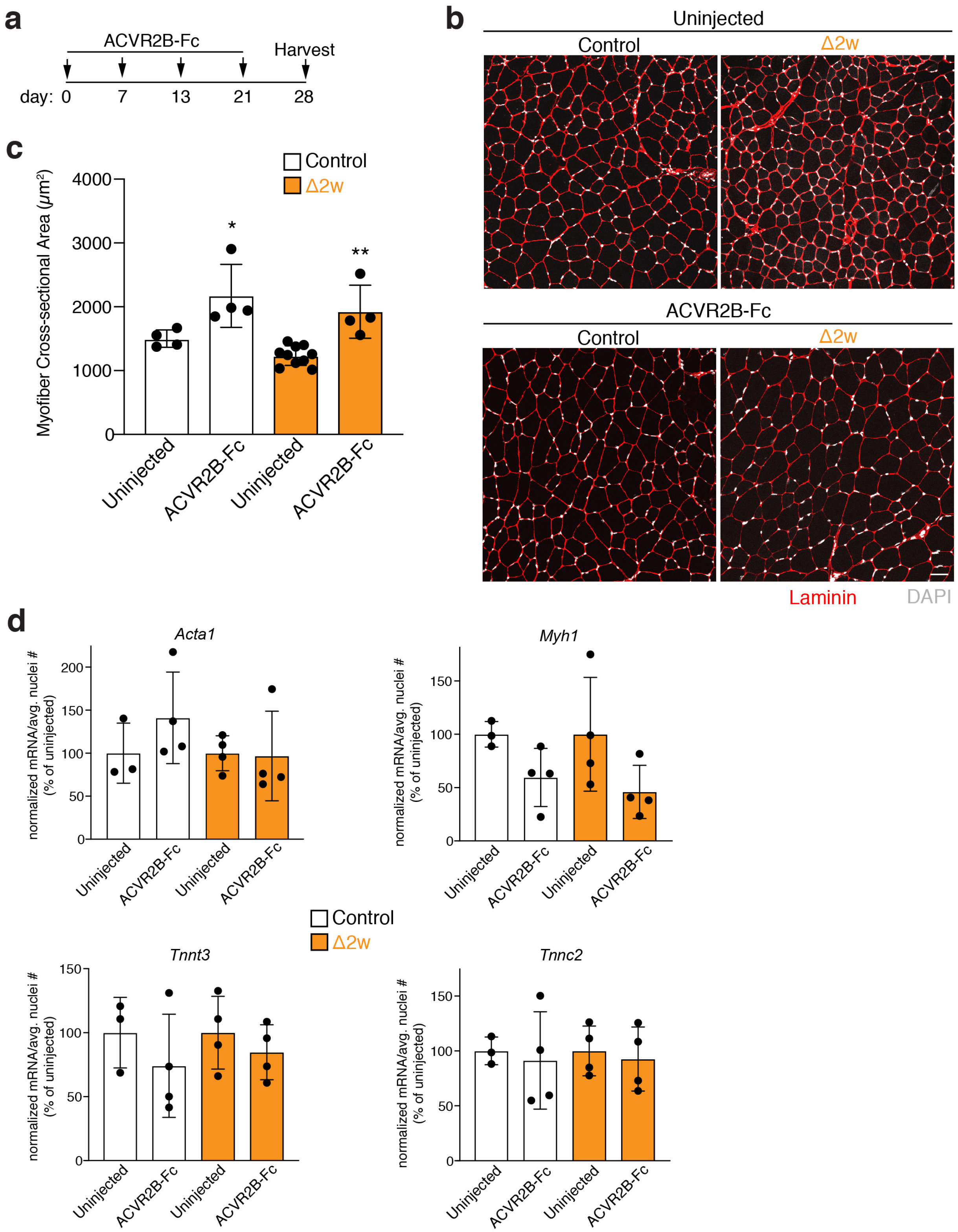

Supplementary Fig. 6 A stimulus that increases myofiber volume does not elicit changes in mRNA concentrations. **a** Experimental design used to inhibit myostatin signaling. ACVR2B-Fc was administered to 3.5 month old control and Δ2w mice once a week for

four weeks. **b** Representative sections of Δ2w and control tibialis anterior muscle, uninjected (top) and ACVR2B-Fc-treated (bottom). Images show an increase in myofiber size in both groups following ACVR2B-Fc treatment for four weeks. Sections were immunostained with laminin antibodies (red) and stained with DAPI (white). **c** Analysis of cross-sectional area (CSA) using the images represented in (**b**) showed significant increases in the CSA of tibialis anterior muscle fibers in both control and Δ2w muscle injected with ACVR2B-Fc compared to their uninjected controls. At least 3 20x images were analyzed per mouse (n = 4-10 biologically independent animals). **d** Concentration of transcripts coding for key skeletal muscle structural genes (*Acta1*, *Myh1*, *Tnnt3*, and *Tnnc2*) on a per nuclear basis are not significantly increased in control or Δ2w muscle following ACVR2B-Fc treatment. To obtain these values, relative transcript levels were determined by semi-quantitative qPCR from samples, which were then normalized to total RNA and average myonuclear numbers for each genotype (n = 3-4 biologically independent animals). Statistical analyses and data presentation: **c** one-way ANOVA with a Tukey correction for multiple comparisons; *p<0.05, **p<0.01; significance compared to uninjected groups. Data are represented as mean ± SD. Scale bar: 50 µm. Source data are provided as a Source Data file.

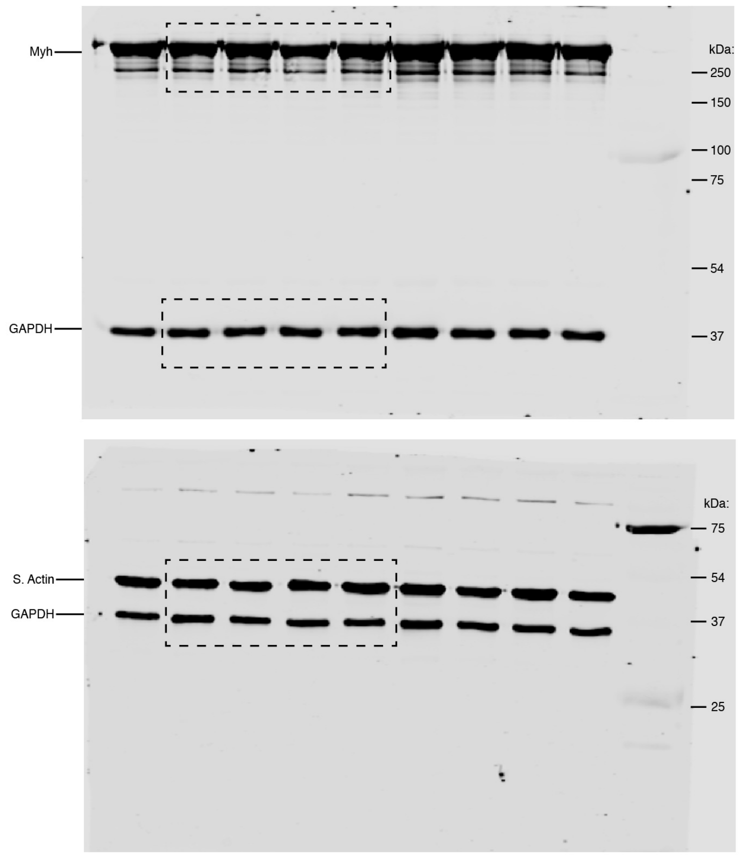

Supplementary Fig. 7 Full western blots for cropped images shown in Fig. 5h.
