# Supplementary material for: Nuclear numbers in syncytial muscle fibers promote size but limit the development of larger myonuclear domains
Source: Nat Commun. 2020 Dec 8;11:6287. doi: 10.1038/s41467-020-20058-7 (PMC7722938; doi:10.1038/s41467-020-20058-7)
Supplement: Supplementary file 3 — Reporting Summary [file 41467_2020_20058_MOESM3_ESM.pdf]

nature research

Corresponding author(s): Douglas P Millay

Last updated by author(s): 08/26/2020

Reporting Summary

Nature Research wishes to improve the reproducibility of the work that we publish. This form provides structure for consistency and transparency in reporting. For further information on Nature Research policies, see Authors & Referees and the Editorial Policy Checklist .

Statistics

For all statistical analyses, confirm that the following items are present in the figure legend, table legend, main text, or Methods section.

n/a	Confirmed	
☐	☒	The exact sample size (*n*) for each experimental group/condition, given as a discrete number and unit of measurement
☐	☒	A statement on whether measurements were taken from distinct samples or whether the same sample was measured repeatedly
☐	☒	The statistical test(s) used AND whether they are one- or two-sided<br>*Only common tests should be described solely by name; describe more complex techniques in the Methods section.*
☐	☒	A description of all covariates tested
☐	☒	A description of any assumptions or corrections, such as tests of normality and adjustment for multiple comparisons
☐	☒	A full description of the statistical parameters including central tendency (e.g. means) or other basic estimates (e.g. regression coefficient) AND variation (e.g. standard deviation) or associated estimates of uncertainty (e.g. confidence intervals)
☐	☒	For null hypothesis testing, the test statistic (e.g. *F*, *t*, *r*) with confidence intervals, effect sizes, degrees of freedom and *P* value noted<br>*Give P values as exact values whenever suitable.*
☒	☐	For Bayesian analysis, information on the choice of priors and Markov chain Monte Carlo settings
☒	☐	For hierarchical and complex designs, identification of the appropriate level for tests and full reporting of outcomes
☒	☐	Estimates of effect sizes (e.g. Cohen's *d*, Pearson's *r*), indicating how they were calculated

Our web collection on statistics for biologists contains articles on many of the points above.

Software and code

Policy information about availability of computer code

Data collection	Dynamic Muscle Control (DMC v5.5) (Aurora Scientific)<br>Nikon A1R confocal microscope<br>Nikon SpectraX widefield microscope
Data analysis	Dynamic Muscle Analysis (DMA v5.3) (Aurora Scientific)<br>Panther Classification System<br>NIS Elements software (Nikon) 5.20.00 build 1423<br>Imaris software (Bitplane) 9.20<br>GraphPad Prism 8<br>Microsoft Excel 16.4<br>FIJI 2.0.0-rc-64/1.52e

For manuscripts utilizing custom algorithms or software that are central to the research but not yet described in published literature, software must be made available to editors/reviewers. We strongly encourage code deposition in a community repository (e.g. GitHub). See the Nature Research guidelines for submitting code & software for further information.

Data

Policy information about availability of data

All manuscripts must include a data availability statement. This statement should provide the following information, where applicable:

- Accession codes, unique identifiers, or web links for publicly available datasets
- A list of figures that have associated raw data
- A description of any restrictions on data availability

The datasets generated during the current study are available at NCBI's Gene Expression Omnibus database (GSE141296). Other reagents are available from the authors. Data availability statement is in the manuscript.

Field-specific reporting

Please select the one below that is the best fit for your research. If you are not sure, read the appropriate sections before making your selection.

☒ Life sciences ☐ Behavioural & social sciences ☐ Ecological, evolutionary & environmental sciences

For a reference copy of the document with all sections, see nature.com/documents/nr-reporting-summary-flat.pdf

Life sciences study design

All studies must disclose on these points even when the disclosure is negative.

Sample size	No sample size calculation was performed prior to experiments. We initially chose sample size based on previous experience and the sample size is sufficient to achieve statistical significance at the reported effect sizes. This was confirmed with a post-hoc power analysis.
Data exclusions	No data were excluded from the analyses.
Replication	Experiments were performed on multiple cohorts of mice, with control mice in each cohort. All attempts of replication were successful.
Randomization	Mice were randomly placed into experimental groups.
Blinding	Investigators were not blinded because the samples are easily distinguished due to the genetic perturbation resulting in obvious reductions in myonuclear number.

Reporting for specific materials, systems and methods

We require information from authors about some types of materials, experimental systems and methods used in many studies. Here, indicate whether each material, system or method listed is relevant to your study. If you are not sure if a list item applies to your research, read the appropriate section before selecting a response.

Materials & experimental systems

n/a	Involved in the study	
☐	☒	Antibodies
☒	☐	Eukaryotic cell lines
☒	☐	Palaeontology
☐	☒	Animals and other organisms
☒	☐	Human research participants
☒	☐	Clinical data

Methods

n/a	Involved in the study	
☒	☐	ChIP-seq
☒	☐	Flow cytometry
☒	☐	MRI-based neuroimaging

Antibodies

Antibodies used	MYH7, Developmental Studies Hybridoma Bank, cat. # (and clone name): BA-D5<br>MYH2, Developmental Studies Hybridoma Bank, cat. # (and clone name): SC-71<br>MYH4, Developmental Studies Hybridoma Bank, cat. # (and clone name): BF-F3<br>Rabbit anti-laminin, Sigma-Aldrich, cat. # L9393-.2ML<br>MF20, Developmental Studies Hybridoma Bank<br>Sarcomeric actin, Sigma, A2172<br>Gapdh, Fitzgerald, 10R-G109A
Validation	All antibodies used have been validated in previous work in the field.

Animals and other organisms

Policy information about studies involving animals; ARRIVE guidelines recommended for reporting animal research

Laboratory animals	Mouse, Myomaker loxP/loxP and Pax7-CreER mice are on the C57Bl6 strain. Both male and female mice were used. Mice were used from postnatal day 0 through 5 months of age.
Wild animals	There are no wild animals used in this study.
Field-collected samples	There are no field collected samples.
Ethics oversight	All animal procedures were approved by Cincinnati Children's Hospital Medical Center's Institutional Animal Care and Use Committee.

Note that full information on the approval of the study protocol must also be provided in the manuscript.
